# Hemosiderin-Laden Macrophages in Canine Mammary Carcinomas

**DOI:** 10.3390/ani13233634

**Published:** 2023-11-24

**Authors:** Giada Giambrone, Cecilia Vullo, Roberto Puleio, Claudia Rifici, Stefania Di Giorgio, Alessandra Sfacteria

**Affiliations:** 1 Department of Veterinary Sciences, University of Messina, Via G. Palatucci, 98168 Messina, Italy; giada.giambrone@studenti.unime.it (G.G.); claudia.rifici@unime.it (C.R.); alessandra.sfacteria@unime.it (A.S.); 2 Department of Chemical, Biological, Pharmaceutical and Environmental Sciences, University of Messina, Viale Ferdinando Stagno d’Alcontres 31, 98166 Messina, Italy; cecilia.vullo@unime.it; 3 Histopathology and Immunohistochemistry Laboratory, Istituto Zooprofilattico Sperimentale della Sicilia, Via Gino Marinuzzi 3, 90129 Palermo, Italy; roberto.puleio@izssicilia.it

**Keywords:** dog, mammary gland, neoplasm, immunohistochemistry, HLMs, iron, VEGF, CD204

## Abstract

**Simple Summary:**

Macrophages play a key role in cancer. The aim of this study was to identify hemosiderin-laden macrophages (HLMs) and speculate their possible role in canine mammary carcinoma. HLMs, which were variously localized in cancer peritumoral and stromal areas, expressed CD204, an M2 macrophagic marker. In addition, they showed positivity for angiogenic and protumoral molecules, such as VEGF or TGF-α. Therefore, HLMs need to be considered for a possible role in cancer progression and survival in canine mammary tumors, protecting cells from hypoxia.

**Abstract:**

Macrophages are among the main actors in cancer immunoediting, with several functions, including recycling iron and packaging it in hemosiderin. Even though TAMs are widely studied in breast cancer and canine mammary tumors, hemosiderin-laden macrophages (HLMs) have not received as much attention. Considering the growing interest in iron metabolism in cancer, this study aims to evaluate the presence of HLMs in canine mammary tumors. Fifty cases of canine mammary carcinomas presenting aggregates of pigmented macrophages were chosen. Prussian blue and Meguro staining were performed to assess the presence of iron. Immunohistochemistry was carried out to try to identify macrophagic phenotypes and hypothesize their role. Evaluation of the H&E sections showed that pigmented macrophages were variously localized in peritumoral and stromal areas. These pigmented cells were variably stained with Prussian blue and reacted strongly with DAB in the Meguro staining method, thus confirming the presence of iron within them. In their immunohistochemistry, the HLMs were negative for the MAC387 but positive for CD 204 and VEGF. Considering their positivity for CD 204, HLMs could be M2 macrophages that supply iron to both the neoplastic cells and the tumor inflammatory microenvironment, promoting angiogenesis and protecting cancer cells from hypoxia.

## 1. Introduction

The hypothesis of cancer immunoediting, which is at present widely accepted, postulates that the immune system and cancer are strictly connected and influence tumor growth. This process comprises three phases: elimination, equilibrium, and escape. In the elimination phase, the immune system identifies and destroys the transformed cells recognized as dangerous for the organism. If some potentially tumorigenic cells survive, they enter into the next stage of equilibrium, in which the cancer cells are kept dormient. In the last phase, the tumor growth becomes invisible to the immune surveillance [1,2,3]. Therefore, the tumor is strongly related to its stromal microenvironment, which is a dynamic entity characterized by the involvement of different chemokines and immune cell populations in which several actors interact with the tumor cells, and among them, macrophages play a key role. 

Macrophages are the main components of the mononuclear phagocyte system and are virtually present in all tissues in an organism. Their number increases further in inflammation, wounding, and malignancy [4]. They are a plastic cell type capable of reacting to microenvironmental signals [5]. Macrophages are among the first responders during inflammation, producing cytokines such as IL-1β, IL-6, and TNF-α to recruit more macrophages and leukocytes to mount an inflammatory response [6]. However, the extensive release of inflammatory cytokines is harmful to tissue repair, and the damaged cells release other cytokines, such as IL-4 and IL-13. These signals polarize macrophages that induct processes like angiogenesis and basement membrane remodeling [7,8]. Based on these two functions, macrophages are classified as proinflammatory macrophages, or M1, and anti-inflammatory macrophages, or M2, but these pathways can converge with one another [9,10].

Increasing numbers of studies on macrophages have demonstrated that these two phenotypes are also present in tumors, with two different functions: M1 macrophages promote tumor resolution, and M2 macrophages propagate tumorigenesis [11]. The M2 cells, the most representative subtype of tumor-associated macrophages (TAMs), contribute to tumor growth, producing molecules that result in basement membrane breakdown and deposition, angiogenesis, recruitment of other inflammatory populations as lymphocytes, and overall immune suppression [12,13,14]. Specifically, their proangiogenic role is already known in relation to VEGF (vascular endothelial growth factor) production and VEGFR1 (VEGF receptor 1) expression [15]. In addition, M2 macrophages are the main producers of TGF-α (transforming growth factor alpha), a molecule functionally related to the epidermal grow factor (EGF) family, with a potent mitogen action for epithelial cells and fibroblasts and an angiogenic role [16]. Moreover, TAMs seem to be involved in a paracrine loop in the tumor microenvironment, whereby tumor-released factors activate p53 (a member of the transcription factor family that also includes p63 and p73) in local macrophages, in turn driving them to express cytokines that may further promote tumorigenesis, potentially both through direct effects on the tumor and by promoting the infiltration of other inflammatory cells [17].

Another important role for macrophages is in iron homeostasis. Iron is an essential element for all organisms, and it is required for different metabolic pathways, such as DNA-synthesis, hematopoiesis, and oxygen transport [18]. Iron metabolism comprises the steps of uptake, storage, and release. Macrophages are involved in recycling iron from the hemoglobin of senescent erythrocytes [19,20]; iron is further accumulated in crystalline, granular ferritin aggregates called hemosiderin, leading to the name of hemosiderin-laden macrophages (HLMs) [21]. To regulate iron homeostasis, macrophages have evolved multiple pathways to acquire, recycle, process, store, and transport iron [19]. Considering the two different macrophagic phenotypes, it is not surprising that they are differentially involved in the iron homeostasis mechanism. M1 cells sequester iron, while M2 cells provide recycled iron to their microenvironment [22]. Indeed, during infections, M1 macrophages sequester iron to avoid its use by invading pathogens [23]. At the same time, these macrophages acquire an iron sequestration phenotype in tumor microenvironments to prevent tumor progression [19]. 

In inflammatory diseases, M2 cells increase the expression of scavenging receptors such as CD163 and CD91, recognizing and clearing dead cells to prevent an inappropriate activation of host inflammatory cells and to control excessive tissue damage during wound healing [24]. In addition, these receptors avoid the toxic effects of extracellular hemoglobin accumulating during hemolysis and inflammation [25]. Furthermore, M2 macrophages increase the level of ferroportin, an iron transporter involved in cellular iron release [26,27]. Additionally, iron export favors hypoxia-inducible factor 1 (HIF-1) expression in macrophages [28]. Indeed, iron deficiency induces HIF and its downstream targets, which are part of the M2 cell gene signature [29]. In TAMs, a high ferroportin-mediated iron release is found, causing higher iron availability in the tumor microenvironment, which enhances tumor growth and proliferation [19].

The presence and the prognostic value of TAMs are largely studied in human medicine in different tumors such as breast cancer. Some studies demonstrate that the density of M2 macrophages is positively correlated with the grade and the malignancy of breast cancer, representing a poor prognostic element [30,31]. Similarly, TAMs have also been researched in canine mammary tumors, and they seem to be related to more aggressive histotypes with lymph node metastasis and poor prognosis [32,33,34,35]. 

On the other side, the role of HLMs is still poorly investigated in mammary tumors. Iron metabolism has received a growing interest in breast and other cancers, considering its possible implications for chemotherapy [36,37,38,39,40]. However, the significance of hemosiderin deposits in tumors is poorly studied. In breast cancer, HLMs are beginning to be evaluated in vivo through the use of iron magnetic resonance imaging (MRI), which could also be employed in the future to map and quantify the size of these deposits [21]. 

Considering the growing interest in iron in cancer, this study aims to evaluate the presence of HLMs in canine mammary carcinomas. Moreover, an M2 phenotype is suggested, along with a hypothesis of the possible role of HLMs in tumor growth and proliferation processes and also in relation to mast cells, which are already recognized to have a proangiogenic role in the tumor microenvironment. 

## 2. Materials and Methods

Fifty cases of malignant canine mammary tumors with pigmented macrophages were retrieved from the archives of the Unit of Veterinary Pathology of the Department of Veterinary Sciences of Messina. All the cases were reclassified according to the most recent classification [41]. We included in the study 20 cases of simple carcinomas, 20 of non-simple carcinomas, and 10 ductal-associated carcinomas, as reported in Table 1. The neoplastic histotypes were then assigned to a grade of malignancy according to the histological criteria provided by Peña et al. [42], with 50% being grade 1, 38% grade 2, and 12% grade 3 carcinomas (Table 1). 

The pigmented macrophages were identified on hematoxylin and eosin (H&E) sections, and their localization was evaluated. For this study, pigmented macrophages located near hemorrhagic areas and blood extravasations were not considered. They were assessed as stromal or peritumoral following the indication already given by Salgado et al. [43] for the localization of TILs (tumor-infiltrating lymphocytes). Briefly, peritumoral HLMs were in the periphery of tumoral masses, while stromal HLMs were dispersed in the stroma between the carcinoma cells and not directly in contact with carcinoma cells. Total HLMs were obtained from the sum of stromal and peritumoral HLMs. Total, stromal, and peritumoral pigmented macrophages were counted. The correlations between number of HLMs and histological subtype and histological tumor grade were determined with the Kruskal–Wallis test using Jamovi computer software (version 2.3—https://www.jamovi.org/, accessed on 21 October 2023). The correlations among total and stromal HLMs of each pair of histological subtypes were determined using the Dwass–Steel–Critchlow–Fligner (DSCF) post hoc test [44]. Statistical significance was based on a 5% (0.05) significance level.

In the same cases, Prussian blue staining and a modified Perls method, also called Meguro staining, were performed [45]. For the Meguro stain, briefly, formalin-fixed, paraffin-embedded sections were deparaffinized and rehydrated. The sections were then incubated with Perls reagent (2% HCl mixed 1:1 with 2% potassium ferrocyanide) for 30 min in a humid chamber at 60 °C. After incubation, endogenous peroxidase was inhibited by incubating with freshly prepared 3% hydrogen peroxide for 30 min at room temperature. Staining was developed in 3,3′-diaminobenzidine (DAB) without counterstain. Additionally, to avoid possible false positives caused by the similar natural color of pigmented macrophages and DAB, we performed Meguro staining using Vector VIP as the developing system. Specifically, Vector VIP, as DAB, is a peroxidase-conjugated chromogen producing a purple color instead of brown. Liver sections were included as positive controls. To be sure that the coloration was really related to the binding of the chromogen with Prussian blue crystals in Meguro stain, negative controls were inserted by omitting the Perls solution.

Immunohistochemistry was then performed on the same samples with antibodies reported in Table 2. Samples were fixed in formalin and embedded in paraffin. Then, 5 µm sections on poly-L-lysine-coated slides underwent antigen unmasking by means of microwave steaming in citrate buffer at pH 6 or EDTA at pH 8. Hydrogen peroxide was used to block the endogenous peroxidases, extending the reaction time to 30 min to bleach the natural color of pigmented macrophages as much as possible. Blocking reagent was used to block non-specific protein reactions (ChemCruz—UltraCruz Blocking Reagent, Santa Cruz Biotechnology, Dallas, TX, USA). The sections were then incubated with the selected primary antibodies overnight at 4 °C. As a revelation method, biotin-conjugated secondary antibodies (as reported in Table 2) were then applied, followed by an incubation with acetylated Streptavidin (Biospa antibiotic products, Milan, Italy; for biotin-conjugated antibodies only). Vector Vip or DAB enzyme substrate, followed by nuclear counterstain with Hematoxylin (Carazzi’s Hemalum), were used to stain the immune reaction. 

Each round of IHC included negative reagent controls and internal positive controls. Each antibody was tested under different conditions, e.g., probing samples with and without antigen retrieval (AR), substituting biotin detection system with HRP-conjugated secondary antibodies. The use of multiple primary antibodies of the same isotype and similar concentrations represented a set of irrelevant negative reagent controls. Immunohistochemistry positivity was assessed as membranous, cytoplasmatic, or nuclear for the different antibodies, as reported in Table 2.

## 3. Results

Among the selected carcinomas, various localizations of pigmented macrophages were detected. On hematoxylin-eosin stain, they had a finely speckled, brownish cytoplasmic granular staining in some cases, while in others, they also contained larger yellow-brown crystals. The number and distribution varied between the different histotypes (simple, non-simple, and ductal-associated carcinomas) as well as between the different grades, as shown in Table 3.

- Total HLMs

Total HLMs were more represented in tubulopapillary (129 ± 71) and tubular carcinomas (107 ± 59). Complex carcinomas showed the smallest number of total HLMs (29 ± 15). Even if the results of the Kruskal–Wallis test comparison of total HLMs with the histological subtype were statistically significant (*p* = 0.04), the post hoc evaluation test was not.

- Stromal HLMs

Carcinoma and malignant myoepithelioma showed more stromal HLMs than the other histotypes (79 ± 31). Tubulopapillary carcinomas showed a similar result in stromal HLMs but with great variability within the selected cases (77 ± 78). Stromal HLMs were not detected in anaplastic carcinoma, and they were rarely detected in complex carcinomas (8 ± 7). 

The results of the Kruskal–Wallis test comparison of stromal HLMs with the histological subtype were statistically significant (*p* = 0.002). The post hoc DSCF test showed significant results for the comparisons of carcinoma arising in complex adenoma/benign mixed tumors and complex carcinoma and for ductal carcinoma and complex carcinoma.

- Peritumoral HLMs

Peritumoral localization was registered in all histotypes, and the anaplastic carcinoma had the highest value (89). The correlation between peritumoral HLMs and the histotypes was not statistically relevant. 

Total HLMs were higher in grade I carcinoma, while stromal HLMs were less represented in grade III carcinoma. Nonetheless, a correlation between total, stromal, and peritumoral HLMs and histological grade was not found. 

In some tumors, such as tubular carcinoma (grade I), tubulopapillary carcinoma (grade I), carcinoma and malignant myoepithelioma (grade II), and ductal carcinoma (grade I and II), pigmented macrophages were located immediately abutting the neoplastic epithelium (intraepithelial HLMs) (Table 4). Another localization was near areas with atypical peritumoral dysplasia in all histotypes (except for the anaplastic carcinoma) and carcinomas of different grades. These results are summarized and schematized in Table 4. 

Isolated groups were near areas of necrosis of the neoplastic epithelium.

**Figure 1:**
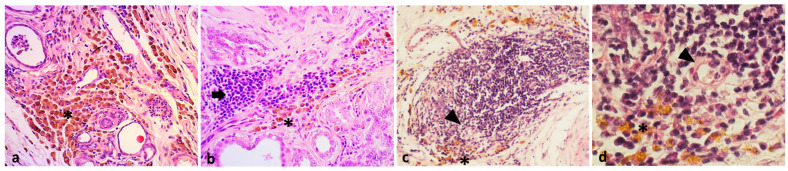
(**a**) Brown-pigmented macrophages (asterisk) in a peritumoral area of grade I tubular carcinoma (H&E, 20×); (**b**) pigmented macrophages (asterisk) in the marginal area of stromal TILs (arrow) in a grade II tubulopapillary carcinoma (H&E, 20×); (**c**) pigmented macrophages (asterisk) in marginal area of a TLS with HEVs (arrowhead) in a grade III solid carcinoma (H&E, 10×); (**d**) details of figure (**c**) with pigmented macrophages (asterisk) and HEV (arrowhead) in a TLS (H&E, 40×).

In addition, a typical spatial disposition in relation to other inflammatory populations in the tumor microenvironment was detected. HLMs were often intermingled or in the marginal area of relevant lymphocytic infiltrates (TILs). A marginal arrangement was also found around cell aggregates recognized as tertiary lymphoid structures (TLSs) where the inflammatory cells showed a prevalent follicular arrangement composed of plasma cells, lymphocytes, and macrophages near to high endothelial venules (HEVs) (Figure 1).

**Figure 2:**
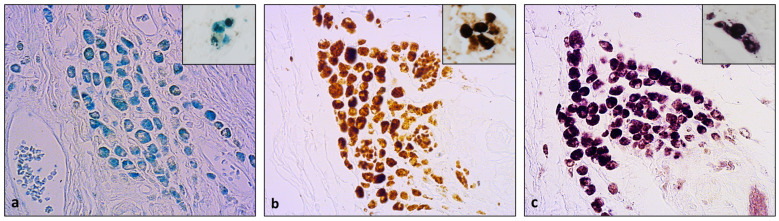
(**a**) Blue staining with Prussian blue in pigmented macrophages in canine mammary carcinoma (Prussian blue, 40×) and positive control (inset, Prussian blue, 60×); (**b**) pigmented macrophages exhibiting brown color with Meguro stain (Meguro stain, 40×) and positive control (inset, Meguro stain, 60×); (**c**) purple color in pigmented macrophages with modified Meguro stain (modified Meguro stain, 40×) and positive control (inset, modified Meguro stain, 60×).

Pigmented macrophages stained to a pronounced blue with the Prussian blue stain. The stain was cytoplasmic, with different densities and intensities amongst the pigmented macrophages and within the same cell (Figure 2a). The presence of the iron deposits was further assessed with the Meguro stain, which gave a pronounced brown staining with different intensities of the granules within the cell and amongst the cells in the same group (Figure 2b). Upon performing modified Meguro staining with Vector VIP, the iron deposits were revealed as purple granules of different intensities and distributions, as already assessed with the former stains (Figure 2c). Similar results were obtained in the liver sections. The HLMs in the liver were prevalently presented as isolated cells. They acquired a blue staining with Prussian blue, both with a granular appearance and, more frequently, as large blue crystals (Figure 2a, inset). The Meguro stain gave an intense brown color to HLMs, similarly to the pigmented macrophages in mammary tumors (Figure 2b, inset). The HLMs in the liver were stained purple with the modified Meguro stain (Figure 2c, inset).

**Figure 3:**
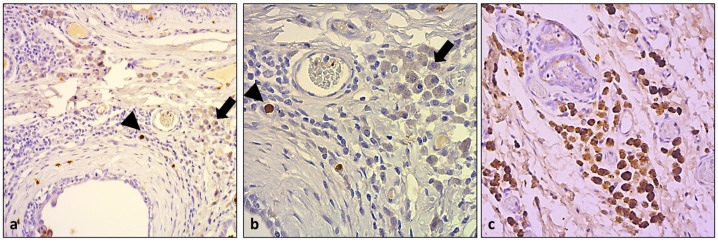
(**a**) Macrophage marker MAC387 highlighted scattered macrophages (arrowhead), but HLMs (arrow) were negative for the same antibody (DAB, 10×); (**b**) details of figure (**a**) (DAB, 40×); (**c**) HLMs were strongly positive for CD204 (DAB, 20×).

Immunohistochemical investigation of the macrophage marker MAC387 highlighted a scattered positivity of macrophages in both the stromal and peritumoral locations and within the TLSs, but none of the HLMs were positive (Figure 3a,b). Instead, these expressed a strongly cytoplasmatic and finely granular positivity for CD 204 (Figure 3c). This was also expressed by other scattered macrophages near the tumor or in the TLSs.

**Figure 4:**
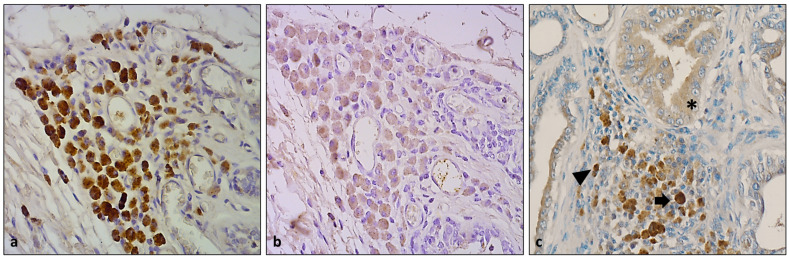
(**a**) HLM positivity for VEGF (DAB, 40×); (**b**) negative control (DAB, 40×); (**c**) VEGF expressed in HLMs (arrow), mast cells (arrowhead), and neoplastic epithelium (asterisk) (DAB, 20×).

The HLMs showed a cytoplasmatic positivity for VEGF, with a uniform, densely granular cytoplasmic and membranous distribution (Figure 4). VEGF was also expressed in the vascular endothelium and in the neoplastic epithelium and also in other inflammatory cell populations, such as mast cells. 

**Figure 5:**
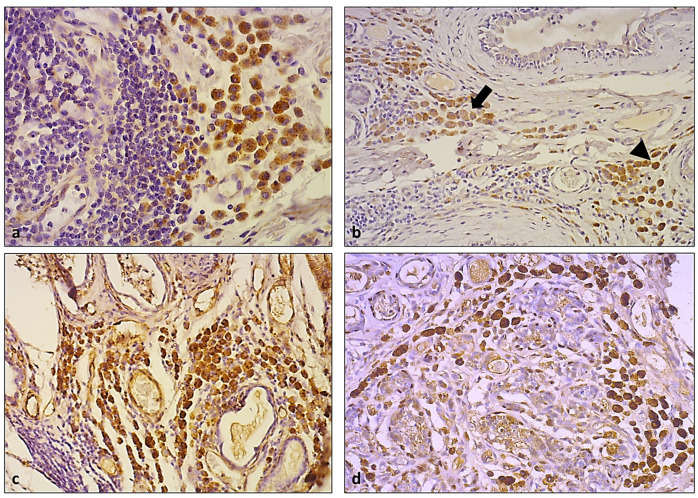
(**a**) HLMs were positive for VEGFR, especially near TILs or TLS (DAB, 40×); (**b**) mast cell tryptase was detected in mast cells (arrowhead) and in some cytoplasmatic granules in HLMs (arrow) (DAB, 10×); (**c**) TGF-α positivity in numerous HLMs and endothelial cells (DAB, 20×); (**d**) p63 positivity in HLMs and in nuclei of myoepithelial cells (DAB, 20×).

VEGFR localization was similar to VEGF, with a major intensity and distributions in HLMs located near the TILs and TLSs (Figure 5a).

Mast cell tryptase was expressed in variously located mast cells. In the most conspicuous groups of HLMs, interspersed mast cells were highlighted. Furthermore, some of those identified as HLMs showed a slight, granular positivity for tryptase along the margin of the cytoplasm (Figure 5b).

TGFα showed a homogeneous positivity in all HLMs detected. A slight positivity was also detected in the vascular endothelial cells (Figure 5c). Additionally, HLMs and myoepithelial cells and some epithelial cells were positive for p63 (Figure 5d).

## 4. Discussion

The role of macrophages in recycling iron is well known, and HLMs are always identified in the liver and spleen. Their role in neoplasia is poorly understood. In breast cancer, HLMs are described, but the studies focus mainly on their clinical implications for chemotherapy and imaging with iron MRI [21,46,47,48]. Marques et al. [49] described the presence of iron in macrophages located in the stromal area of canine and feline mammary tumors. However, the research was focused more on the expression of iron-related proteins than macrophage subsets and suggested no differences between benign and malignant tumors. 

The present study included only canine malignant tumors, focusing on the characterization of HLMs. The H&E results showed that the presence and localization of brown-pigmented macrophages varied between tumors and were not related to tumor grading. Regarding the histotypes, only total and stromal HLMs showed statical differences (*p* = 0.04 and *p* = 0.002, respectively). However, the post hoc evaluation was not significant for the total HLMs, which is probably linked to the weakly significant p-value. For the stromal HLMs, the post hoc DSCF test showed significant results for the comparisons of complex carcinoma with carcinoma arising in complex adenoma/benign mixed tumors and with ductal carcinoma. Indeed, stromal HLMs were less represented in complex carcinoma. However, some of the groups involved in the study had small caseloads, leading to a loss of statistical power. Therefore, in this study, a correlation between stromal HLMs and canine mammary histotypes can only be speculated. Further investigations are ongoing to increase the number of cases per histotype and verify the statical significance of stromal HLMs.

Prussian blue stain confirmed the presence of iron-ferric ions, which are commonly released from hemosiderin and ferritin in acid solutions. Meguro stain in both variants emphasized the amount of iron in these cells [45].

Macrophage marker MAC387 has been considered a panmacrophagic marker for years in veterinary medicine [50,51]. However, recently, some studies evidenced that this protein is expressed only by a small percentage of the macrophages in the tumor microenvironment. Cells expressing MAC387 are also positive to Iba1, a recognized marker for M1 macrophagic phenotype. In addition, it seems that MAC387 is specifically related to newly infiltrating tissue macrophages [52,53]. 

In the present study, HLMs were negative for the macrophage marker MAC387 but strongly positive for CD204. Also called Scavenger Receptor-A, CD204 is a transmembrane protein highly expressed in M2-polarized macrophages [54,55]. Higher numbers of CD204-positive TAMs are associated with a worse prognosis in different tumors in both human and veterinary medicine [53,56,57]. Specifically, Parisi et al. [33] showed that M2-polarized TAMs are correlated with more aggressive histological subtypes and higher grades of canine mammary carcinomas, lymphatic invasion, and poorer survival, confirming the potential of CD204 protein as a prognostic factor. 

These results are in contrast with a study by Leftin et al. [21] where HLMs were not polarized to either phenotypic extreme in mice mammary tumors. On the contrary, in lung metastasis, HLMs were recognized as M2 cells, while in brain metastasis, they were predominantly M1 macrophages [21].

On the basis of their immunopositivity to CD204, VEGF, VEGFR-1, and TGF-α, in this study, HLMs can be assumed to be M2-polarized macrophages, producing substances that are helpful for cancer. VEGF is one of the most important molecules used by tumors during the escape phases. VEGF promotes angiogenesis, stimulating the proliferation and survival of endothelial cells and increasing vessel permeability [58]. In canine mammary tumors, VEGF data has been statistically correlated with intratumoral microvessel density, and both measures were greater in less-differentiated malignant neoplasms, demonstrating that angiogenesis and malignancy increase together [59]. 

HEVs develop through VEGF signals. HEVs are normally located in lymphoid organs. However, they can also develop in cancer and permit inflammatory cells to arrive in tumor sites [60]. Inflammatory cells can be organized in conspicuous groups of only lymphocytes (TILs) or in more complex structures of mixed populations, simulating lymphoid tissues (TLSs). In canine mammary tumors, as in breast cancer, TLSs can be described with different patterns of organization and are characterized by the presence of HEVs [61]. In this study, conspicuous groups of HLMs were located in the marginal areas of TILs or TLSs and were positive for VEGF, suggesting that they also have an angiogenic role in the development of HEVs. Indeed, Virmani et al. [62] already showed that the presence of HLMs is positively correlated with angiogenesis in atherosclerotic lesions in human coronary arteries. 

Additionally, the protumoral angiogenic role of HLMs could also be related to their location near necrotic neoplastic epithelium. Indeed, upregulation of VEGF could be linked to the hypoxic condition present in the necrotic compartments [63]. 

VEGF activates different receptors, among them VEGFR-1, promoting cancer proliferation [64]. The axis of VEGF/VEGFR-1 is also able to recruit monocytes and macrophages, promoting the development of TAMs [65,66]. Therefore, the same HLMs could be involved in recruiting other M2 macrophages.

High levels of VEGR-1 have been identified in metastases-associated macrophages (MAMs) in breast cancer [67]. In this study, HLMs were variably positive for VEFGR-1. Nevertheless, the greater positivity was concentrated near TLSs. In a previous study, we found TLSs were often located near or around vessels with emboli, so we hypothesized that they could be involved in the metastatic process, as already reported in human medicine [61,68]. In the same way, HLMs could have a potential metastatic role.

VEGF was also expressed by mast cells, which were detected by mast cell tryptase antibody and are already recognized for their angiogenic role in cancer [69]. Mast cells were scattered in the stroma but also intermingled in conspicuous groups of HLMs. At the same time, some cytoplasmatic granules in the HLMs were positive for mast cell tryptase. A hypothesis could be that the HLMs phagocytized the molecules from degranulated mast cells. Nevertheless, a more probable conclusion could be that mast cells interact with macrophages to polarize them. Indeed, mast cell tryptase is already recognized for converting macrophages into an M2 subtype (M2a) involved in wound healing and fibrosis [70]. Therefore, mast cells and macrophages could cooperate in tumors to regulate the inflammatory microenvironment to guarantee tumor survival.

Increasing the evidence of a protumoral role, HLMs expressed a strong positivity for TGF-α. TGF-α is one of the several growth factors that are expressed in both normal and malignant mammary epithelial cells, and macrophages are the principal productors [71,72,73]. However, interaction with different cells and molecules can result in different roles in the neoplastic tissue [72]. 

HLMs also expressed p63. This is an important regulator of cell proliferation and survival, with a major role in the maintenance of stem cells and their differentiation, and it is also involved in the carcinogenesis of many cell types [74]. In mammary glands, p63 is considered a sensitive and specific myoepithelial marker [75]. In addition, p63 has different functions in iron metabolism, with mechanisms that are still unknown [76]. Being a member of the p53 family, p63 could be involved in mechanisms of tumorigenesis and in promoting the infiltration of additional inflammatory cells, modeling the tumor microenvironment, as already demonstrated for p53 [17]. Moreover, p73, another member of the p53 family, has been recognized to be directly involved in the mechanisms of M2 macrophage polarization [77]. Therefore, it is possible that p63 is involved in this matter. However, further investigations should be conducted to better understand the role of its expression in HLMs.

Considering their positivity for CD204 and their expression of angiogenic and protumoral molecules, this study suggests that HLMs could be considered a new subtype of M2 macrophages.

In this study, we only included tumors presenting pigmented macrophages. In many cases, pigmented macrophages were not detected. 

A possible explanation for this absence could be related to the integrity of the hormonal axis in dogs. Estrogens, specifically, are considered pivotal for mammary cancer growth both in women and dogs, and one of the recognized pathways involves redox cycling of estrogen metabolites. Indeed, these molecules can damage DNA by generating superoxide, which can participate in iron-dependent Fenton reactions to generate DNA-damaging hydroxyl radicals [78]. Many cases in this paper were from intact dogs, and additional studies are ongoing to increase the caseload and assess if a correlation exists.

Additionally, low iron in tissue may stimulate HIF-1α activation and consequently promote angiogenesis, while high iron may increase oxidative stress. Both these situations promote breast tumor growth [79]. At the same time, estrogens can increase the production of TGF-α [72].

Another feasible explanation could be correlated to hypoxia. The hypoxic condition induces the production of a broad array of stimulating factors and recruits M2-polarized macrophages that express high levels of HIF. This regulator activates different pathways to recall other macrophages, induces the synthesis of angiogenic substances, and increases the release of nutrients for tumors, among them iron [19,80]. 

This last hypothesis could explain the location of the HLMs near necrotic areas or near preneoplastic lesions, where all the neoplastic autonomous mechanisms of survival and growth are probably not yet active.

## 5. Conclusions

Macrophages are highly plastic cells that can play different roles in neoplasia. To date, HLMs are still understudied in the tumor context. The results of this study highlight how HLMs may represent a subtype of M2 macrophages that is probably directly involved in tumor growth and proliferation. Given the growing interest in iron and its implication in tumorigenesis and tumor progression, HLMs should be given more attention. Indeed, these could represent an additional point of contact between canine mammary tumors and breast cancer, considering that the former have long been proposed as a model for translational study of the latter. All of this also opens the possibility of identifying new diagnostic and predictive markers and even new therapeutic targets.

## Figures and Tables

**Table 1:** Cases included in the study.

Major Group	N. Cases	Histotypes	N. Cases Grade I	N. Cases Grade II	N. Cases Grade III
Simple carcinoma	8	Tubular carcinoma	5	3	-
	6	Tubulopapillary carcinoma	3	3	-
	5	Solid carcinoma	-	1	4
	1	Anaplastic carcinoma	-	-	1
Non-simple carcinoma	10	Carcinoma arising in complex adenoma/benign mixed tumor	10	-	-
	5	Complex carcinoma	2	3	-
	5	Carcinoma and malignant myoepithelioma	1	3	1
Ductal-associated carcinoma	10	Ductal carcinoma	4	6	-

N. cases = number of cases.

**Table 2:** Primary and secondary antibodies used for HIC.

Antibody I	Clone	Type of Antibody I	Target	Dilution	Brand	Positive Staining Pattern
Macrophage marker	MAC387	Mouse monoclonal	Macrophages	1:200	Santa Cruz Biotechnology	Cytoplasmatic
MSR1/SCARA1/CD204		Rabbit monoclonal	Macrophages	1:200	Sino Biological, Beijing, China	Cytoplasmatic/ Membranous
VEGF	VG-1	Mouse monoclonal	VEGF	1:50	Santa Cruz Biotechnology	Cytoplasmatic/ Membranous
VEGFR 1	VEGFR 1	Rabbit polyclonal	VEGF receptor 1	1:100	GeneTex, Inc., Irvine, CA, USA	Cytoplasmatic/ Membranous
Mast cell Tryptase	AA1	Mouse monoclonal	Mast cell	1:100	Santa Cruz Biotechnology	Cytoplasmatic
TGF-α	Ab-3 (Cocktail)	Mouse monoclonal	TGF-α	1:100	NeoMarkers, Portsmouth, NH, USA	Cytoplasmatic
p63	D-9	Mouse monoclonal	p63	1:100	Santa Cruz Biotechnology	Nuclear/ Cytoplasmatic
**Antibody II**		**Antibody type**		**Dilution**		**Brand**
Goat anti-mouse IgG-B		Mouse IgG		1:100		BioSpa, Milan
Goat anti-rabbit IgG-B		Rabbit IgG		1:200		BioSpa, Milan

**Table 3:** Comparison of total, stromal, and peritumoral HLMs for histotypes and grading.

	N. Cases	Total HLMs		Stromal HLMs		Peritumoral HLMs	
		Mean ± SD	*p*	Mean ± SD	*p*	Mean ± SD	*p*
Histotype			0.04		0.002 ^a,b^		NS
Tubular carcinoma	8	107 ± 59		67 ± 45		40 ± 24	
Tubulopapillary carcinoma	6	129 ± 71		77 ± 78		52 ± 43	
Solid carcinoma	5	58 ± 34		14 ± 20		43 ± 28	
Anaplastic carcinoma	1	89		0		89	
Carcinoma arising in complex adenoma/benign mixed tumor	10	91 ± 31		58 ± 27		33 ± 22	
Complex carcinoma	5	29 ± 15		8 ± 7		21 ± 11	
Carcinoma and malignant myoepithelioma	5	93 ± 49		79 ± 31		14 ± 22	
Ductal carcinoma	10	85 ± 44		47 ± 22		38 ± 27	
Histological grade			NS		NS		NS
Grade I	25	96 ± 51		57 ± 45		39 ± 29	
Grade II	19	83 ± 54		53 ± 42		29 ± 24	
Grade III	6	67 ± 28		18 ± 20		48 ± 35	

^a^ = carcinoma arising in complex adenoma/benign mixed tumor vs. complex carcinoma significant in post hoc Dwass–Steel–Critchlow–Fligner test; ^b^ = ductal carcinoma vs. complex carcinoma significant in post hoc Dwass–Steel–Critchlow–Fligner test; N. cases = number of cases; SD = standard deviation; *p* = *p*-value; NS = not significant.

**Table 4:** Histotypes and grades of canine mammary carcinomas presenting intraepithelial HLMs and HLMs near atypical peritumoral dysplasia.

	Histotype	Grade	N. Cases
Intraepithelial HLMs	Tubular carcinoma	I	1
	Tubulopapillary carcinoma	I	1
	Carcinoma and malignant myoepithelioma	II	2
	Ductal carcinoma	I	2
		II	1
HLMs near atypical peritumoral dysplasia	Tubular carcinoma	I	3
		II	1
	Tubulopapillary carcinoma	I	2
	Solid carcinoma	III	1
	Carcinoma arising in complex adenoma/benign mixed tumor	I	4
	Complex carcinoma	I	1
	Carcinoma and malignant myoepithelioma	II	1
		III	1
	Ductal carcinoma	I	2
		II	1

N. cases = number of cases.

## Data Availability

Data are contained within the article.

## References

[1] Lasek W. (2022). Cancer immunoediting hypothesis: History, clinical implications and controversies. Cent. Eur. J. Immunol..

[2] Schreiber R.D., Old L.J., Smyth M.J. (2011). Cancer immunoediting: Integrating immunity’s roles in cancer suppression and promotion. Science.

[3] Dunn G.P., Bruce A.T., Ikeda H., Old L.J., Schreiber R.D. (2002). Cancer immunoediting: From immunosurveillance to tumor escape. Nat. Immunol..

[4] Hume D.A. (2006). The mononuclear phagocyte system. Curr. Opin. Immunol..

[5] Okabe Y., Medzhitov R. (2014). Tissue-specific signals control reversible program of localization and functional polarization of macrophages. Cell.

[6] Malyshev I., Malyshev Y. (2015). Current Concept and Update of the Macrophage Plasticity Concept: Intracellular Mechanisms of Reprogramming and M3 Macrophage “Switch” Phenotype. Biomed. Res. Int..

[7] Martinez F.O., Helming L., Gordon S. (2009). Alternative activation of macrophages: An immunologic functional perspective. Annu. Rev. Immunol..

[8] Van Dyken S.J., Locksley R.M. (2013). Interleukin-4- and interleukin-13-mediated alternatively activated macrophages: Roles in homeostasis and disease. Annu. Rev. Immunol..

[9] Italiani P., Boraschi D. (2014). From Monocytes to M1/M2 Macrophages: Phenotypical vs. Functional Differentiation. Front. Immunol..

[10] Murray P.J., Allen J.E., Biswas S.K., Fisher E.A., Gilroy D.W., Goerdt S., Gordon S., Hamilton J.A., Ivashkiv L.B., Lawrence T. (2014). Macrophage activation and polarization: Nomenclature and experimental guidelines. Immunity.

[11] Ngambenjawong C., Gustafson H.H., Pun S.H. (2017). Progress in tumor-associated macrophage (TAM)-targeted therapeutics. Adv. Drug Deliv. Rev..

[12] Wang H.W., Joyce J.A. (2010). Alternative activation of tumor-associated macrophages by IL-4: Priming for protumoral functions. Cell Cycle.

[13] Quail D.F., Joyce J.A. (2013). Microenvironmental regulation of tumor progression and metastasis. Nat. Med..

[14] Caux C., Ramos R.N., Prendergast G.C., Bendriss-Vermare N., Ménétrier-Caux C. (2016). A Milestone Review on How Macrophages Affect Tumor Growth. Cancer Res..

[15] Cassetta L., Pollard J.W. (2018). Targeting macrophages: Therapeutic approaches in cancer. Nat. Rev. Drug Discov..

[16] Schreiber A.B., Winkler M.E., Derynck R. (1986). Transforming growth factor-alpha: A more potent angiogenic mediator than epidermal growth factor. Science.

[17] Lowe J.M., Menendez D., Fessler M.B. (2014). A new inflammatory role for p53 in human macrophages. Cell Cycle.

[18] Crichton R.R., Wilmet S., Legssyer R., Ward R.J. (2002). Molecular and cellular mechanisms of iron homeostasis and toxicity in mammalian cells. J. Inorg. Biochem..

[19] Jung M., Mertens C., Brüne B. (2015). Macrophage iron homeostasis and polarization in the context of cancer. Immunobiology.

[20] De Back D.Z., Kostova E.B., van Kraaij M., van den Berg T.K., van Bruggen R. (2014). Of macrophages and red blood cells; a complex love story. Front. Physiol..

[21] Leftin A., Ben-Chetrit N., Klemm F., Joyce J.A., Koutcher J.A. (2017). Iron imaging reveals tumor and metastasis macrophage hemosiderin deposits in breast cancer. PLoS ONE.

[22] Cairo G., Recalcati S., Mantovani A., Locati M. (2011). Iron trafficking and metabolism in macrophages: Contribution to the polarized phenotype. Trends Immunol..

[23] Nairz M., Schroll A., Sonnweber T., Weiss G. (2010). The struggle for iron—A metal at the host-pathogen interface. Cell Microbiol..

[24] Biswas S.K., Mantovani A. (2010). Macrophage plasticity and interaction with lymphocyte subsets: Cancer as a paradigm. Nat. Immunol..

[25] Buehler P.W., D’Agnillo F., Schaer D.J. (2010). Hemoglobin-based oxygen carriers: From mechanisms of toxicity and clearance to rational drug design. Trends Mol. Med..

[26] Donovan A., Lima C.A., Pinkus J.L., Pinkus G.S., Zon L.I., Robine S., Andrews N.C. (2005). The iron exporter ferroportin/Slc40a1 is essential for iron homeostasis. Cell Metab..

[27] Recalcati S., Locati M., Marini A., Santambrogio P., Zaninotto F., De Pizzol M., Zammataro L., Girelli D., Cairo G. (2010). Differential regulation of iron homeostasis during human macrophage polarized activation. Eur. J. Immunol..

[28] Semenza G.L. (2007). Hypoxia-inducible factor 1 (HIF-1) pathway. Sci. STKE.

[29] Takeda N., O’Dea E.L., Doedens A., Kim J.W., Weidemann A., Stockmann C., Asagiri M., Simon M.C., Hoffmann A., Johnson R.S. (2010). Differential activation and antagonistic function of HIF-{alpha} isoforms in macrophages are essential for NO homeostasis. Genes Dev..

[30] Mahmoud S.M., Lee A.H., Paish E.C., Macmillan R.D., Ellis I.O., Green A.R. (2012). Tumour-infiltrating macrophages and clinical outcome in breast cancer. J. Clin. Pathol..

[31] Medrek C., Pontén F., Jirström K., Leandersson K. (2012). The presence of tumor associated macrophages in tumor stroma as a prognostic marker for breast cancer patients. BMC Cancer.

[32] Monteiro L.N., Dos Reis D.C., Salgado B.S., Cassali G.D. (2021). Clinical significance and prognostic role of tumor-associated macrophages infiltration according to histologic location in canine mammary carcinomas. Res. Vet. Sci..

[33] Parisi F., Tesi M., Millanta F., Gnocchi M., Poli A. (2021). M1 and M2 tumour-associated macrophages subsets in canine malignant mammary tumours: An immunohistochemical study. Res. Vet. Sci..

[34] Monteiro L.N., Rodrigues M.A., Gomes D.A., Salgado B.S., Cassali G.D. (2018). Tumour-associated macrophages: Relation with progression and invasiveness, and assessment of M1/M2 macrophages in canine mammary tumours. Vet. J..

[35] Sfacteria A., Napoli E., Rifici C., Commisso D., Giambrone G., Mazzullo G., Marino G. (2021). Immune Cells and Immunoglobulin Expression in the Mammary Gland Tumors of Dog. Animals.

[36] Buss J.L., Greene B.T., Turner J., Torti F.M., Torti S.V. (2004). Iron chelators in cancer chemotherapy. Curr. Top. Med. Chem..

[37] Heath J.L., Weiss J.M., Lavau C.P., Weschler D.S. (2013). Iron deprivation in cancer—Potential therapeutic implications. Nutrients.

[38] Kalinowski D.S., Richardosn D.R. (2005). The evolution of iron chelators for the treatment of iron overlaod disease and cancer. Pharmacol. Rev..

[39] Le N.T., Richardson D.R. (2002). The role of iron in cell cycle progression and the proliferation of neoplastic cells. Biochim. Biophys. Acta.

[40] Torti S.V., Torti F.M. (2013). Iron and cancer: More ore to be mined. Nat. Rev. Cancer.

[41] Zappulli V., Pena L., Rasotto R., Goldschmidt M.H., Gama A., Scruggs J.L., Kiupel M. (2018). Surgical Pathology of Tumors of Domestic Animals: Volume 2: Mammary Tumors.

[42] Peña L., De Andrés P.J., Clemente M., Cuesta P., Pérez-Alenza M.D. (2013). Prognostic value of histological grading in noninflammatory canine mammary carcinomas in a prospective study with two-year follow-up: Relationship with clinical and histological characteristics. Vet. Pathol..

[43] Salgado R., Denkert C., Demaria S., Sirtaine N., Klauschen F., Pruneri G., Wienert S., Van den Eynden G., Baehner F.L., Penault-Llorca F. (2015). The evaluation of tumor-infiltrating lymphocytes (TILs) in breast cancer: Recommendations by an International TILs Working Group 2014. Ann. Oncol..

[44] Critchlow E.D., Fligner A.M. (1991). On distribution-free multiple comparisons in the one-way analysis of variance. Commun. Stat.-Theory Methods.

[45] Meguro R., Asano Y., Odagiri S., Li C., Iwatsuki H., Shoumura K. (2007). Nonheme-iron histochemistry for light and electron microscopy: A historical, theoretical and technical review. Arch. Histol. Cytol..

[46] Sato A., Yumi M., Sakai T., Gomi N., Horii R.R., Akiyama F., Iwase T., Ohno S. (2017). A case of breast Cancer with hemosiderin deposits resembling calcifications on mammography. Jpn. J. Breast Cancer.

[47] Shamama S., Shazima S., Flora D.L., Waseemoddin P., Abhishek S.N., Mubeen K. (2018). Therapy-induced histopathological changes in breast cancers: The changing role of pathology in breast Cancer diagnosis and treatment. Cancer Transl. Med..

[48] Harada T.L., Nakashima K., Uematsu T., Sugino T., Nishimura S., Takahashi K., Tadokoro Y., Hayashi T., Watanabe J., Nakamoto S. (2019). Imaging features of breast cancer with marked hemosiderin deposition: A case report. Eur. J. Radiol. Open.

[49] Marques O., Canadas A., Faria F., Oliveira E., Amorim I., Seixas F., Gama A., Lobo-da-Cunha A., Silva B.M.D., Porto G. (2017). Expression of iron-related proteins in feline and canine mammary gland reveals unexpected accumulation of iron. Biotech. Histochem..

[50] Vanherberghen M., Day M.J., Delvaux F., Gabriel A., Clercx C., Peeters D. (2009). An immunohistochemical study of the inflammatory infiltrate associated with nasal carcinoma in dogs and cats. J. Comp. Pathol..

[51] Silveira T.L., Veloso E.S., Gonçalves I.N.N., Costa R.F., Rodrigues M.A., Cassali G.D., Del Puerto H.L., Pang L.Y., Argyle D.J., Ferreira E. (2020). Cyclooxygenase-2 expression is associated with infiltration of inflammatory cells in oral and skin canine melanomas. Vet. Comp. Oncol..

[52] Soulas C., Conerly C., Kim W.K., Burdo T.H., Alvarez X., Lackner A.A., Williams K.C. (2011). Recently infiltrating MAC387+ monocytes/macrophages. Am. J. Pathol..

[53] Porcellato I., Sforna M., Lo Giudice A., Bossi I., Musi A., Tognoloni A., Chiaradia E., Mechelli L., Brachelente C. (2022). Tumor-Associated Macrophages in Canine Oral and Cutaneous Melanomas and Melanocytomas: Phenotypic and Prognostic Assessment. Front. Vet. Sci..

[54] Komohara Y., Ohnishi K., Kuratsu J., Takeya M. (2008). Possible involvement of the M2 anti-inflammatory macrophage phenotype in growth of human gliomas. J. Pathol..

[55] Heusinkveld M., van der Burg S.H. (2011). Identification and manipulation of tumor associated macrophages in human cancers. J. Transl. Med..

[56] Ichimura T., Morikawa T., Kawai T., Nakagawa T., Matsushita H., Kakimi K., Kume H., Ishikawa S., Homma Y., Fukayama M. (2014). Prognostic significance of CD204-positive macrophages in upper urinary tract cancer. Ann. Surg. Oncol..

[57] Ohtaki Y., Ishii G., Nagai K., Ashimine S., Kuwata T., Hishida T., Nishimura M., Yoshida J., Takeyoshi I., Ochiai A. (2010). Stromal macrophage expressing CD204 is associated with tumor aggressiveness in lung adenocarcinoma. J. Thorac. Oncol..

[58] Sia D., Alsinet C., Newell P., Villanueva A. (2014). VEGF signaling in cancer treatment. Curr. Pharm. Des..

[59] Restucci B., Papparella S., Maiolino P., De Vico G. (2002). Expression of vascular endothelial growth factor in canine mammary tumors. Vet. Pathol..

[60] Karakhanova S., Link J., Heinrich M., Shevchenko I., Yang Y., Hassenpflug M., Bunge H., von Ahn K., Brecht R., Mathes A. (2015). Characterization of myeloid leukocytes and soluble mediators in pancreatic cancer: Importance of myeloid-derived suppressor cells. Oncoimmunology.

[61] Giambrone G., Di Giorgio S., Vullo C., Marino G., Puleio R., Mariotti F., Mazzullo G., Sfacteria A. (2022). Does TLS Exist in Canine Mammary Gland Tumours? Preliminary Results in Simple Carcinomas. Vet. Sci..

[62] Virmani R., Kolodgie F.D., Burke A.P., Finn A.V., Gold H.K., Tulenko T.N., Wrenn S.P., Narula J. (2005). Atherosclerotic plaque progression and vulnerability to rupture: Angiogenesis as a source of intraplaque hemorrhage. Arterioscler. Thromb. Vasc. Biol..

[63] Shweiki D., Itin A., Soffer D., Keshet E. (1992). Vascular endothelial growth factor induced by hypoxia may mediate hypoxia-initiated angiogenesis. Nature.

[64] Bhattacharya R., Ye X.C., Wang R., Ling X., McManus M., Fan F., Boulbes D., Ellis L.M. (2016). Intracrine VEGF Signaling Mediates the Activity of Prosurvival Pathways in Human Colorectal Cancer Cells. Cancer Res..

[65] Incio J., Tam J., Rahbari N.N., Suboj P., McManus D.T., Chin S.M., Vardam T.D., Batista A., Babykutty S., Jung K. (2016). PlGF/VEGFR-1 Signaling Promotes Macrophage Polarization and Accelerated Tumor Progression in Obesity. Clin. Cancer Res..

[66] Ceci C., Atzori M.G., Lacal P.M., Graziani G. (2020). Role of VEGFs/VEGFR-1 Signaling and its Inhibition in Modulating Tumor Invasion: Experimental Evidence in Different Metastatic Cancer Models. Int. J. Mol. Sci..

[67] Qian B.Z., Zhang H., Li J., He T., Yeo E.J., Soong D.Y., Carragher N.O., Munro A., Chang A., Bresnick A.R. (2015). FLT1 signaling in metastasis-associated macrophages activates an inflammatory signature that promotes breast cancer metastasis. J. Exp. Med..

[68] Sofopoulos M., Fortis S.P., Vaxevanis C.K., Sotiriadou N.N., Arnogiannaki N., Ardavanis A., Vlachodimitropoulos D., Perez S.A., Baxevanis C.N. (2019). The prognostic significance of peritumoral tertiary lymphoid structures in breast cancer. Cancer Immunol. Immunother..

[69] Sfacteria A., Lanteri G., Grasso G., Macrì B., Mazzullo G. (2011). Mast cells in canine mammary gland tumour: Number, distribution and EPOR positivity. Vet. Comp. Oncol..

[70] White M.J., Gomer R.H. (2015). Trypsin, Tryptase, and Thrombin Polarize Macrophages towards a Pro-Fibrotic M2a Phenotype. PLoS ONE.

[71] Lippman M.E., Dickson R.B., Gelmann E.P., Rosen N., Knabbe C., Bates S., Bronzert D., Huff K., Kasid A. (1987). Growth regulation of human breast carcinoma occurs through regulated growth factor secretion. J. Cell. Biochem..

[72] Ciardiello F., Kim N., McGeady M.L., Liscia D.S., Saeki T., Bianco C., Salomon D.S. (1991). Expression of transforming growth factor alpha (TGF alpha) in breast cancer. Ann. Oncol..

[73] Sun J., Cui H., Gao Y., Pan Y., Zhou K., Huang J., Lan J., Wei Q., Liu X., Liu L. (2018). TGF-α Overexpression in Breast Cancer Bone Metastasis and Primary Lesions and TGF-α Enhancement of Expression of Procancer Metastasis Cytokines in Bone Marrow Mesenchymal Stem Cells. Biomed. Res. Int..

[74] Galoczova M., Coates P., Vojtesek B. (2018). STAT3, stem cells, cancer stem cells and p63. Cell. Mol. Biol. Lett..

[75] Stefanou D., Batistatou A., Nonni A., Arkoumani E., Agnantis N.J. (2004). p63 expression in benign and malignant breast lesions. Histol. Histopathol..

[76] Shen J., Sheng X., Chang Z., Wu Q., Wang S., Xuan Z., Li D., Wu Y., Shang Y., Kong X. (2014). Iron metabolism regulates p53 signaling through direct heme-p53 interaction and modulation of p53 localization, stability, and function. Cell Rep..

[77] Rozenberg J.M., Zvereva S., Dalina A., Blatov I., Zubarev I., Luppov D., Bessmertnyi A., Romanishin A., Alsoulaiman L., Kumeiko V. (2021). Dual Role of p73 in Cancer Microenvironment and DNA Damage Response. Cells.

[78] Torti S.V., Torti F.M. (2013). Cellular iron metabolism in prognosis and therapy of breast cancer. Crit. Rev. Oncog..

[79] Jian J., Yang Q., Dai J., Eckard J., Axelrod D., Smith J., Huang X. (2011). Effects of iron deficiency and iron overload on angiogenesis and oxidative stress-a potential dual role for iron in breast cancer. Free Radic. Biol. Med..

[80] He Z., Zhang S. (2021). Tumor-Associated Macrophages and Their Functional Transformation in the Hypoxic Tumor Microenvironment. Front. Immunol..

